# Single, but not dual, attack by a biotrophic pathogen and a sap-sucking insect affects the oak leaf metabolome

**DOI:** 10.3389/fpls.2022.897186

**Published:** 2022-08-03

**Authors:** Laura J. A. van Dijk, Emilia D. E. Regazzoni, Benedicte R. Albrectsen, Johan Ehrlén, Ahmed Abdelfattah, Hans Stenlund, Katharina Pawlowski, Ayco J. M. Tack

**Affiliations:** ^1^Department of Ecology, Environment and Plant Sciences, Stockholm University, Stockholm, Sweden; ^2^Department of Plant Physiology, Umeå Plant Science Centre, Umeå University, Umeå, Sweden; ^3^Institute of Environmental Biotechnology, Graz University of Technology, Graz, Austria

**Keywords:** *Erysiphe alphitoides*, GC-MS, metabolomics, pedunculate oak, plant-pathogen-insect interactions, powdery mildew, *Quercus robur*, *Tuberculatus annulatus*

## Abstract

Plants interact with a multitude of microorganisms and insects, both below- and above ground, which might influence plant metabolism. Despite this, we lack knowledge of the impact of natural soil communities and multiple aboveground attackers on the metabolic responses of plants, and whether plant metabolic responses to single attack can predict responses to dual attack. We used untargeted metabolic fingerprinting (gas chromatography-mass spectrometry, GC-MS) on leaves of the pedunculate oak, *Quercus robur*, to assess the metabolic response to different soil microbiomes and aboveground single and dual attack by oak powdery mildew (*Erysiphe alphitoides*) and the common oak aphid (*Tuberculatus annulatus*). Distinct soil microbiomes were not associated with differences in the metabolic profile of oak seedling leaves. Single attacks by aphids or mildew had pronounced but different effects on the oak leaf metabolome, but we detected no difference between the metabolomes of healthy seedlings and seedlings attacked by both aphids and powdery mildew. Our findings show that aboveground attackers can have species-specific and non-additive effects on the leaf metabolome of oak. The lack of a metabolic signature detected by GC-MS upon dual attack might suggest the existence of a potential negative feedback, and highlights the importance of considering the impacts of multiple attackers to gain mechanistic insights into the ecology and evolution of species interactions and the structure of plant-associated communities, as well as for the development of sustainable strategies to control agricultural pests and diseases and plant breeding.

## Introduction

Plants interact with many types of organisms, both below- and aboveground. While some of these organisms are beneficial to the plant, such as mutualistic microorganisms, others attack plants and cause biotic stress, such as herbivores or pathogens. Biotic interactions affect plant chemistry, for example *via* impacts on nutrient acquisition and induction of defenses, causing shifts in the metabolic profile of the plant (Sardans et al., 2021). While previous studies have explored the effects that soil microbial communities (Badri et al., 2013; Ristok et al., 2019; Huberty et al., 2020), single aboveground attackers (e.g., Kant et al., 2004; Marti et al., 2013; Zhang et al., 2020), and dual aboveground attackers (Ponzio et al., 2017) have on plant metabolomes, we lack insights into the importance of soil microbiomes and multiple aboveground attackers in shaping the plant metabolome. Moreover, the vast majority of previous research has focused on a rather limited set of agricultural crops and model plant species (e.g., Sutter and Müller, 2011; Badri et al., 2013; Balmer et al., 2013; Schweiger et al., 2014b; Zhou et al., 2018), while relatively few studies have focused on the responses of natural plant systems where plants are involved in a large number of interactions (Schweiger et al., 2014a; Huberty et al., 2020; Rodrigues et al., 2021). Understanding the changes in plant chemistry in response to interactions with belowground microbial communities and multiple aboveground attackers under experimental conditions is important to predict spatiotemporal variation in plant chemistry in natural settings, as well as its consequences for the outcome of species interactions in nature (van Dam and van der Meijden, 2011).

Soil microorganisms interact with the plant at the rhizosphere, and a range of studies have shown that single microbial species (Zhou et al., 2018) and taxa (Schliemann et al., 2008; Schweiger et al., 2014a) can affect plant metabolomes at the leaf level by colonization of the roots and rhizosphere. For example, species of arbuscular mycorrhizal fungi can facilitate the supply of photosynthates to the fungus by affecting the primary metabolism of their host plant (Schliemann et al., 2008; Schweiger et al., 2014a; Kaur and Suseela, 2020). When broadening the scope from single species or taxa to diverse communities, Badri et al. (2013) demonstrated that differences in soil microbiomes can also lead to differences in leaf metabolomes. Yet, we know comparatively little about the relative importance of belowground microbiomes and other external factors, such as aboveground attackers, in shaping the leaf metabolome (Ristok et al., 2019; Huberty et al., 2020).

Similarly to soil microorganisms, aboveground attackers may affect the chemistry of their host plant in various ways. For example, pathogens or insect herbivores can directly affect plant metabolism *via* the extraction of nutrients, or by interfering with metabolic processes, like photosynthesis or stomatal conductance (Rabbinge et al., 1983; Macedo et al., 2003; Copolovici et al., 2014). Attackers may also affect the plant metabolome *via* induction of defense signaling, such as the salicylic (SA) pathway, which is mostly effective against sap-sucking insects and biotrophic pathogens, and the jasmonic acid (JA) pathway, which is mostly effective against chewing insects and necrotrophic pathogens (Glazebrook, 2005). The defenses induced by SA- or JA- signaling alter the metabolic profile of the plant, and while some studies investigated metabolic responses to dual attack (Ponzio et al., 2017), most studies have focused on single attacks (e.g., Sutter and Müller, 2011; Marti et al., 2013), or on the effects of simulated attacks (e.g., Schweiger et al., 2014b; Papazian et al., 2019). For example, simultaneous induction of the SA- and JA-pathway—which mimics a scenario with multiple attackers on a plant—resulted in non-additive metabolic changes, possibly due to co-regulation of metabolites or reciprocal antagonism between these pathways (Schweiger et al., 2014b). Yet, no study to date has focused on dual attacks by organisms that putatively induce the same defense-related pathways, which might result in additive effects on plant chemistry due to higher overlap in the induction of downstream pathways. Since changes in plant primary and secondary metabolites may affect the performance of plants as well as their attackers, we need to understand the impact of attackers on host plant metabolism to make predictions on the outcomes of interactions between plants, pathogens and herbivores (Johnson et al., 2003; Kang et al., 2018).

Our overarching aim was to explore changes in the leaf metabolome in response to differences in soil microbiomes, as well as in response to aboveground organisms in terms of single or dual attacks by a biotrophic pathogen and a sap-sucking insect. We focused on seedlings of the pedunculate oak *Quercus robur*, two natural soil microbiomes, oak powdery mildew *Erysiphe alphitoides*, and the common oak aphid *Tuberculatus annulatus*. To characterize metabolic changes in response to interactions with soil microorganisms and aboveground attackers, we used gas chromatography-mass spectrometry (GC-MS). We addressed two main questions:

i. How is the leaf metabolome of oak seedlings affected when growing with two different natural soil microbiomes?
ii. What is the effect of single and dual attacks by aphids and powdery mildew on the leaf metabolome of oak seedlings? Can the metabolic response to dual attack be predicted from the responses to single attacks?

Since we previously observed that differences in soil microbiomes affected plant growth (van Dijk et al., in revision), we expected that differences in soil microbiomes would also affect the metabolome of oak leaves. We also expected that aboveground attackers would influence the oak leaf metabolome, and that metabolic changes in response to single attacks would be less pronounced than those upon dual attack. As aphids and powdery mildew putatively induce the same defense pathway (SA) (Thaler et al., 2012; Copolovici et al., 2014), we expected an additive, i.e., predictable, effect of dual attack on the oak leaf metabolome.

## Materials and methods

### Study system

The pedunculate oak *Quercus robur* is a deciduous tree that occurs throughout Europe (Ducousso and Bordacs, 2004), with its northern limit in central Sweden and Norway (Eaton et al., 2016) and its southern limit in central Spain (Petit et al., 2002). The pedunculate oak is host to a diverse community of pathogens and insect herbivores (Southwood et al., 2004). The most common pathogen is oak powdery mildew, *Erysiphe alphitoides* (Desprez-Loustau et al., 2018). *E. alphitoides* is presumably of Asian origin, and was first recorded in Europe in the early twentieth century (Mougou et al., 2008; Desprez-Loustau et al., 2011). Oak powdery mildew is a specialist biotrophic pathogen that grows epiphytically on the leaves, only penetrating the plant epidermis with its feeding structures, the haustoria (Marçais and Desprez-Loustau, 2014). Infection by powdery mildew limits the photosynthetic ability of plants, and the pathogen uses large amounts of photo-assimilates to grow (Edwards and Ayres, 1981; Hajji et al., 2009). During the growing season, oak powdery mildew produces asexual, wind-dispersed spores (conidia), and structures with sexual spores (chasmothecia) are formed at the end of the growing season to allow for overwintering (Bushnell, 2002). Oaks are also attacked by various insect herbivores, such as the specialist common oak aphid *Tuberculatus annulatus*, which feeds on the phloem sap. Overwintering eggs are laid on oak buds and hatch in spring. Aphids reproduce asexually throughout the growing season, and sexually at the end of the season (Bohórquez, 1987).

### Collection of soils, plants, powdery mildew, and aphids

To assess if the leaf metabolome of oak seedlings differs when growing with two different soil microbiomes, natural soils were collected from two locations close to Stockholm University, one from a forest with sandy soil (59.364584, 18.049175) and one from a meadow with clay soil (59.368815, 18.049952). A third natural soil was collected from a forest with mixed sandy and clay soil (59.368763, 18.070849), though this soil was only used for preparation, and not for its microbiome (see below).

Acorns were collected from pedunculate oaks (*Quercus robur*) distributed throughout Stockholm during the autumn of 2018 and stored in potting soil to prevent desiccation at + 4°C before being planted. As we strived for natural conditions within the experiment, we collected acorns from a random set of mother trees, and we did not surface sterilize the acorns. This natural variation in genotypes and acorn microbiomes likely increased the variation of leaf metabolic profiles among seedlings, but is unlikely to cause any biases between treatments, and allows for the assessment of general, rather than genotype- or phenotype-specific, responses. Acorns were directly planted in their designated soil microbiome (see below, “*Preparation of the two soil treatments*”). Seedlings were grown in a growth chamber (10 h 20°C: 14 h 18°C, light: dark, with a light intensity of 110 μmol m^–2^ s^–1^ total radiation at ∼50 cm above the acorns, air humidity 65%), and seedlings were included in the experiment once the first leaves (mostly 3–5 leaves) started to develop (^~^9 weeks after planting of the acorns), and were grown for another 3 weeks before infestation by aboveground attackers (Supplementary Figure 1C).

Powdery mildew spores were collected from trees around the Stockholm University area, from leaves with powdery mildew colonies that grew on the adaxial side of the leaf. The collected spores were used to infect oak seedlings in the greenhouse, on which the powdery mildew colonies were maintained. Aphids were collected from the area surrounding Stockholm University in spring 2018 and were maintained on oak seedlings in the greenhouse.

### Preparation of the two soil treatments

To create two soils that only differed in their microbial composition, and not in abiotic components, we sterilized part of both soils by one cycle of autoclaving shortly after collection of the soils, while soils were moist. We then mixed sterilized soil with unsterilized soil of the other type (1:1) (Supplementary Figure 1A). We also added an equal amount of a third natural sterilized soil to both soil mixes, thus obtaining a 1:1:1 mix of three distinct natural soils. Then, pots of 7 × 7 × 18 cm (700 ml) were filled with a layer of nutrient-poor sterilized potting soil (300 ml, Så och pluggjord, SW Horto, Hammenhög, Sweden), a layer of the natural soil mix (125 ml, either with the forest or meadow microbiome), again a layer of nutrient-poor sterilized potting soil (250 ml), and finally a layer of sterilized sand (25 ml). We henceforth refer to these soils as “forest microbiome” and “meadow microbiome.” While the autoclaving procedure may have left some background contamination, the two resulting soil microbiomes remained highly distinctive (see section “Composition of soil microbiomes”).

### The experiment

To examine the influence of soil microbiomes and of single and dual attacks on the leaf metabolome, oak seedlings growing with each of the two distinct soil microbiomes received four different attacker treatments. The attacker treatments included: (1) healthy seedlings that received no attackers; (2) seedlings infected with powdery mildew; (3) seedlings infested by aphids; and (4) seedlings infected and infested simultaneously by powdery mildew and aphids (Supplementary Figure 1). Attacker treatments were applied to seedlings once seedlings had grown for 3 weeks after developing 3–5 leaves and each treatment was applied to four seedlings growing in soil with the forest microbiome and four growing in soil with the meadow microbiome. During the experiment, seedlings were kept in a growth chamber (10 h 20°C: 14 h 18°C, light: dark, with a light intensity of 110 μmol m^–2^ s^–1^ total radiation at ∼25 cm above the plant tops, air humidity 65%). Seedlings were watered by placing a tray filled with water underneath the plant pot, to avoid direct contact between water and the inoculated powdery mildew spores. All seedlings, including control seedlings, were covered by a pollination bag (type 3D.55, PBS international, Scarborough, United Kingdom) to prevent the spread of attackers to other seedlings.

To infect seedlings with powdery mildew, all leaves of a seedling were gently brushed with spores originating from a greenhouse-maintained mildew colony (∼ 1 cm^2^) (Nicot et al., 2002; van Dijk et al., 2020). To infest plants with aphids, five wingless aphid nymphs were transferred onto five randomly selected leaves of a seedling.

Seedling leaves were collected 72 h after exposure to the attacker treatment. This induction duration has been shown to capture plant metabolic changes upon attack (Sutter and Müller, 2011; Schweiger et al., 2014b; Ponzio et al., 2017), which is also true for the metabolic responses of woody plants, which are usually slightly delayed compared to those of herbaceous plants (Agut et al., 2014; Wang et al., 2016). Moreover, regarding the attackers, the time span of 72 h is well past the time it takes for mildew spores to germinate and infect plant tissues (about 24 h) (Celio and Hausbeck, 1998; Nicot et al., 2002). To capture the overall leaf metabolic response to attack (in both directly attacked and other leaves), we excised the distal part of all leaves (approximately one-fourth of the leaf surface) for each seedling (Kim and Verpoorte, 2010). Leaves were harvested of 40 seedlings in total, yielding 20 replicates for each soil microbiome, and 8 replicates for each attacker treatment. At the time of leaf collection, seedlings had been exposed to their soil microbiome for about 12 weeks (Supplementary Figure 1C), a time span that extends well beyond the time it takes for the roots of seedlings to penetrate all layers of soil. Leaf parts were snap-frozen in liquid nitrogen immediately upon harvest, and then stored at −80°C.

### Soil microbiome analyses

We collected soil samples from 15 random pots for each soil community (forest and meadow soils) at the start of the experiment (i.e., 3 weeks after the pots were filled with soil and acorns were planted, Supplementary Figure 1C). We took care to collect samples that were representative of the whole soil community within the pot by using a soil core (diameter 1 cm) that we punched through the soil to a depth of 10 cm. Collected soil samples were carefully mixed and then stored at −20°C. Before further processing of the samples, samples were frozen at −80°C for 2 days, after which we freeze-dried all samples for 4 days. Bacterial and fungal communities in both soils were characterized with amplicon sequencing of the 16S and ITS regions, respectively. A detailed description of sequencing methods and bioinformatics is provided in van Dijk et al. (in revision), as quoted in Supplementary Text 1.

### Metabolomic analyses

Frozen leaf samples were ground into a fine powder in liquid nitrogen using a mortar and pestle. The homogenized plant tissue was weighed using a microbalance (10 ± 3 mg) while being kept completely frozen using liquid nitrogen. The samples were kept at −80°C until they were shipped on dry ice and processed for GC-MS by the Swedish Metabolomics Centre (SMC), Umeå, Sweden.

At SMC, sample preparation for GC-MS was performed according to Gullberg et al. (2004). More specifically, 750 μL of extraction buffer (20/20/60 v/v/v chloroform:water:methanol) including internal standards were added to each sample. The sample was shaken with a tungsten bead in a mixer mill at 30 Hz for 3 min, after which the bead was removed and the sample was centrifuged at 4°C and 14,000 rpm, for 10 min. 200 μL of supernatant were transferred to a microvial and solvents were evaporated.

Derivatization was performed according to Gullberg et al. (2004). More specifically, 30 μL of methoxyamine (15 μg/μL in pyridine) were added to the dry sample and the sample was shaken vigorously for 10 min before being left to react in room temperature. After 16 h, 30 μL of MSTFA and 30 μL of heptane were added and the sample was shaken and left to react for 1 h in room temperature. 30 μL of methyl stearate (15 ng/μL in heptane) were added before analysis (for details on the GC-MS methods, see Supplementary Text 2).

### Statistical analyses

Fungal and bacterial community compositions of the two soil microbiomes were statistically compared using the *adonis2* function of the *vegan* package (Oksanen et al., 2020), in R v.3.6.1 (R Core Team, 2020).

Data was normalized by dividing the peak area of each compound identified by that of the corresponding injection standard, and subsequently dividing the resulting data by the wet weight of plant tissue (in mg). All multivariate statistical investigations (PCA, OPLS-DA) were performed using SIMCA v.17.0.0 (Umetrics, Umeå, Sweden) (Eriksson et al., 2006). For all models, we used mean centering and unit variance-scaling, which ensures that all metabolites exert the same influence on the model regardless of the abundance of the metabolite.

Differences in the metabolic profiles between seedlings with distinct soil microbiomes (across attacker treatments, *n* = 20 per soil microbiome), as well as among seedlings with different attacker treatments (across soil microbiomes, *n* = 8 per treatment), were first visually explored with a principal component analysis (PCA), which is an unsupervised modeling approach (Jackson, 1991). We then conducted orthogonal partial least squares discriminant analysis (OPLS-DA), which is a supervised multivariate statistical discriminant analysis (Bylesjö et al., 2006), to examine differences between the soil treatments, as well as among the four different attacker treatments. To reveal pairwise differences among specific attacker treatments, we fitted OPLS-DA models including only two attacker treatments (referred to as “pairwise OPLS-DA models”): (i) healthy seedlings vs. mildew only, (ii) healthy seedlings vs. aphids only, (iii) healthy seedlings vs. both mildew and aphids, (iv) mildew only vs. both mildew and aphids, and (v) aphids only vs. both mildew and aphids. All five pairwise OPLS-DA models were limited to one predictive and one orthogonal component to facilitate model comparisons, and to minimize the risk of overfitting the models (Ponzio et al., 2017). For multiple pairwise comparisons, we made SUS-plots (shared and unique structures plots), which allow comparison of two pairwise OPLS-DA models that have one shared and one distinct treatment (Wiklund et al., 2008). Following this strategy, we made three SUS-plots: (i) healthy seedlings vs. mildew only, plotted against healthy seedlings vs. aphids only, (ii) mildew only vs. healthy seedlings, plotted against mildew only vs. both mildew an aphids, (iii) aphids only vs. healthy seedlings, plotted against aphids only vs. both mildew and aphids. SUS plots are based on the *p*(corr)-values, which are the loadings of the metabolites scaled as correlation coefficients (ranging from −1 to 1) between the model and the data. For the SUS-plots, the *p*(corr)-values from the predictive components of the two pairwise OPLS-DA models were plotted against each other (Wheelock and Wheelock, 2013). When analyzing an SUS-plot, the metabolites that are in relatively high abundance in the treatment group shared by both models will cluster toward the lower left corner. Similarly, the metabolites that are in relatively high abundance in the distinct treatment group examined in each OPLS-DA model will cluster toward the top right corner. Metabolites that differ in a similar way in both models will therefore be positioned along a diagonal line that extends through the origin from the lower left to the upper right corner.

We interpreted the VIP scores (variable importance in projections) to estimate the contribution of individual metabolites to the separation of the metabolic profiles. Such metabolites are henceforth referred to as “metabolites of special interest” (VIP ≥ 1), and were further examined with linear models using the function *lm* in R v.3.6.1 (R Core Team, 2020). To analyze the effect of experimental treatments on metabolite concentrations, we modeled each metabolite as a function of attacker treatment. In case of a significant treatment effect, we conducted *post hoc* pairwise comparisons with Tukey’s HSD. In the few instances where model residuals were not normally distributed or variances were unequal, we instead conducted a Kruskal-Wallis test, followed by a *post hoc* Dunn test using the function *dunnTest* in the package *FSA* (Ogle et al., 2021).

## Results

### Composition of soil microbiomes

The bacterial and fungal compositions differed between forest and meadow microbiomes, with soil origin explaining 27 and 23% of the variation in bacterial and fungal microbiomes, respectively [*F*_(1, 28)_ = 10.30, *p* = 0.001 and *F*_(1, 28)_ = 8.56, *p* = 0.001, respectively, *n* = 15 per soil microbiome, Supplementary Figure 2]. The forest and meadow microbiomes differed in their bacterial but not fungal species richness [*F*_(1, 28)_ = 5.14, *p* = 0.03 and *F*_(1, 28)_ = 0.67, *p* = 0.42, respectively].

### Effects of soil microbiomes on plant metabolism

We annotated a total of 74 metabolic compounds, including acids, amino acids, fatty acids, phenolic acids, antioxidants, inositols, phytohormones, sugars and carbohydrates, phytosterols, polyphenols, and tannins (Supplementary Table 1).

**FIGURE 1:**
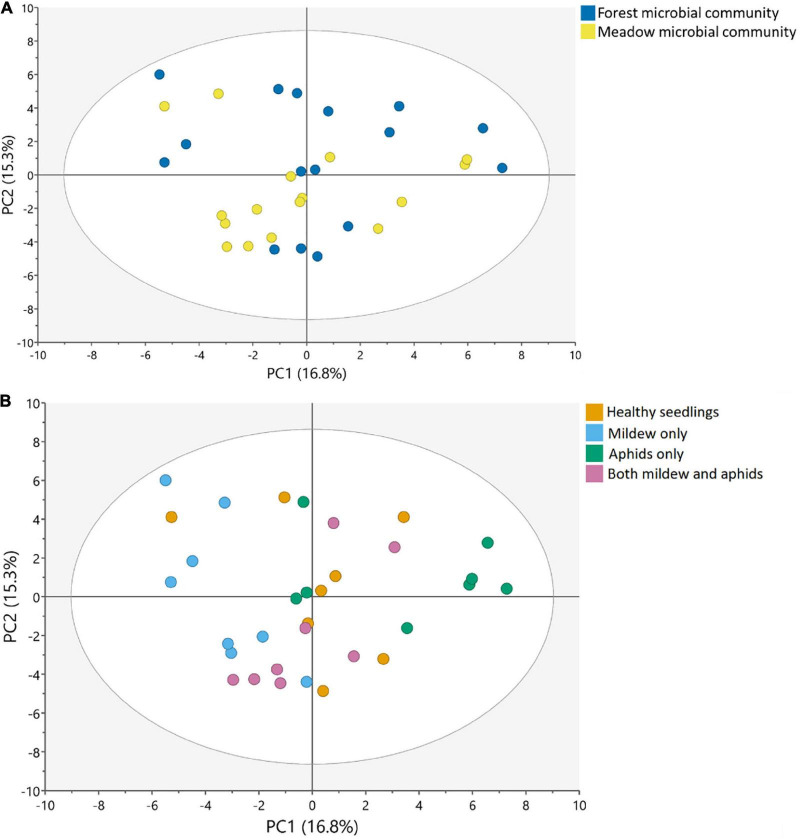
Changes in the metabolic profiles of oak seedlings in response to **(A)** soil microbial communities and **(B)** attacker treatments, visualized in a score plot with the first two components of the PCA model, with the variation explained by the first component on the x-axis, and the variation explained by the second component on the y-axis. **(A)** Colors represent the different soil microbial communities, from forest and meadow, with 20 replicates per soil community (across attacker treatments). **(B)** Colors represent the different attacker treatments, including: (1) Healthy oak seedlings, (2) Seedlings attacked by mildew, (3) Seedlings attacked by aphids and (4) Seedlings attacked by mildew and aphids, with 8 replicates per treatment (across soil microbiomes) (for the loadings of these plots, see Supplementary Figure 6; for a visualization of the OPLS-DA model on attacker treatments, see Supplementary Figure 5).

Seedlings growing with the forest soil microbiome did not differ from seedlings growing with the meadow soil microbiome in leaf metabolic profile (PCA, *n* = 20 per soil microbiome, Figure 1). Differences in the two soil microbiomes could not predict differences in metabolic profiles of oak seedlings (OPLS-DA, cumulative *Q*^2^ = -0.044, Supplementary Table 2).

### Effects of single attack on plant metabolism

**FIGURE 2:**
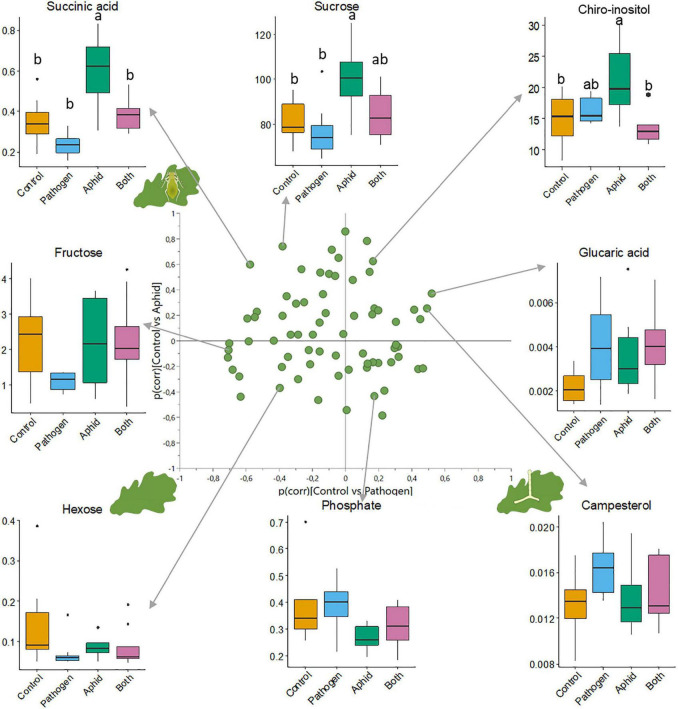
SUS-plot of control vs. pathogen and control vs. aphid with representative boxplots for metabolites at the extremes of the plot. The SUS-plot shows how metabolite levels differ between healthy seedlings and mildew infected seedlings vs. healthy seedlings and aphid infested seedlings. The axes of the SUS-plot are the *p*(corr)-values of each of the two pair-wise OPLS-DA models used in this figure (Supplementary Table 3). The box plots show the response of individual metabolites to attacker treatments, where the vertical axis of the box plots is the relative abundance of the metabolite after normalization. Significant differences between treatments are noted by a letter above the boxes where groups that do not share the same letter differ. There were eight replicates per treatment group, and each treatment is represented by a different color, with control plants as orange, powdery mildew infected plants as blue, aphid infested plants as green, and dual-infected plants as pink.

The leaf metabolic profile of seedlings differed among attacker treatments (Figure 1B, Supplementary Figures 3–5, and Supplementary Table 3). Seedlings attacked by powdery mildew or aphids differed from healthy seedlings, and each attacker left a distinct metabolic imprint, with 24 and 23 metabolites differing between the healthy seedlings and the single attacker treatments (VIP ≥ 1), respectively (Figure 1B and Supplementary Table 4). Many metabolites were uniquely affected by either mildew infection (e.g., upregulation of proline and campesterol, and downregulation of glucose, salicylic acid and pipecolic acid) or aphid infestation (e.g., upregulation of scyllo- and chiro-Inositol, dehydroascorbic acid, ascorbic acid and α-tocopherol), whereas other metabolites were affected by both attackers but in opposite directions (Figure 2, Supplementary Figure 4, and Supplementary Table 4). Sucrose, succinic acid, and malic acid were downregulated in seedlings attacked by powdery mildew, but upregulated in seedlings attacked by aphids, as compared to healthy seedlings (Figure 2, Supplementary Figure 4, and Supplementary Table 4). Only a few metabolites of special interest (arabinose, glucaric acid, and hexose) were influenced in similar directions by mildew and aphid attacks (Figure 2, Supplementary Figure 4, and Supplementary Table 4).

### Effects of dual attack on plant metabolism

In contrast to the distinct metabolic profiles of seedlings experiencing single attacks, dual attack by aphids and powdery mildew resulted in a metabolome which did not significantly differ from that of healthy seedlings (Figure 1B, Supplementary Figures 3–5, and Supplementary Table 3). Only few metabolites tended to differ between healthy oak seedlings and seedlings attacked by both mildew and aphids, e.g., maltotriose, glucaric acid, cellobiose, pipecolic acid, and phenyllactic acid (Supplementary Figures 3B,C, 4).

## Discussion

Our study explored differences in the leaf metabolome of oak seedlings grown with two distinct soil microbiomes and exposed to single or dual aboveground attack. Differences in the selected natural soil microbiomes were not associated with differences in the leaf metabolic profiles of oak seedlings. However, the metabolome differed in response to aboveground attacks by mildew and aphids. Surprisingly though, the metabolome of seedlings that were simultaneously attacked by mildew and aphids could not be distinguished from those of healthy seedlings. Our study suggests that aboveground interactions influence the leaf metabolome and that metabolic responses to multiple aboveground attackers interact. We are the first to study the effects of natural soil microbiomes on the leaf metabolome, and while we did not detect any effects, more studies on a variety of systems, using different methods and studying different time scales, are needed to generalize this result. Our findings highlight the importance of studying plant metabolomes in the context of natural soil communities and multiple attacker species, and contribute to our understanding of the molecular and ecological consequences of plant-microbe-insect interactions in nature.

Our results showed no differences between distinct natural soil microbiomes in their effects on leaf metabolic profiles of oak leaves as determined by GC-MS. This finding is in contrast to previous studies, which have shown that soil microorganisms may influence the plant metabolome, either due to the presence of certain microbial taxa (Schweiger et al., 2014a; Kaur and Suseela, 2020; Guo et al., 2021), or differences in the composition of manipulated microbial communities (Ristok et al., 2019; Huberty et al., 2020). While discrepancies with our study could be due to differences in methods used, another possible explanation could be that the effects of diverse, natural communities might be less pronounced than those of specific microbial taxa or artificial communities. Possibly, in complex microbial communities, plant metabolites are affected in opposite directions by different soil microorganisms, thereby canceling out overall effects on the leaf metabolome (Zuńiga et al., 2017). Besides this, the oak seedlings in our experiment were genetically diverse, and thus the leaf metabolic profiles of plants growing with different soil communities might conceal genotype- or phenotype specific responses (Ossipov et al., 2008). Moreover, acorns in our experiment were likely to possess distinctive microbiomes inherited from the mother tree, which might increase among-replicate variation in our experiment. In contrast to our approach, Badri et al. (2013) focused on a specific ecotype of a model plant species, *Arabidopsis thaliana*, with limited genotypic and phenotypic variation, and found that diverse natural microbial communities influenced the leaf metabolome. Another explanation for the lack of effects from soil microbiomes on the plant metabolome is that soil microbes may have been unable to colonize the rhizosphere during the course of the experiment, though we consider this scenario unlikely given that the roots and soil microbiomes were in direct contact with one another. However, since seedlings were still connected to their acorn, they could have been less responsive to differences in soil microbiomes, since many of the resources are provided by the seed (Long and Jones, 1996). It would be interesting to investigate whether other study systems, with smaller seeds and less maternal resources, would be more responsive to changes in the soil microbiome. At the same time, while we did not detect an overall impact of soil microbiomes on the leaf metabolome, our previous study showed that these two soil communities were associated with differences in oak seedling height after 16 weeks of growing with the soil microbiomes (van Dijk et al., in revision), suggesting that changes in the abundances of a few individual metabolites could still cause different growth responses. To investigate the generality of our findings, and to link the results of mechanistic studies on specific microbial taxa to natural scenarios with diverse microbial communities, we need studies that investigate a multitude of soil microbiomes to be able to correlate the presence of microbial taxa to changes in the metabolome of plants that grow with diverse natural soil microbiomes.

In contrast to the lack of different effects of soil microbiomes, we did detect differences in the leaf metabolomes of oak seedlings upon mildew infection or aphid infestation compared to healthy seedlings. Powdery mildew-infected seedlings showed a decrease in glucose, as well as other sugars and sugar alcohol (sucrose, arabinose, hexose, ribose, erythritol and glucose 1-phosphate). This general decrease in sugars could be related to reduced photosynthetic activity of mildew-infected leaves, or the removal of photosynthates by the pathogen (Hajji et al., 2009; Kurth et al., 2014). In accordance with this, two metabolites involved in the citric acid cycle (malic acid and succinic acid) were downregulated in mildew-infected seedlings, further suggesting an effect of mildew infection on carbon metabolism (Papazian et al., 2016). Regarding defense metabolites in powdery mildew infected seedlings, we observed a decrease of pipecolic acid, a metabolite involved in systemic acquired resistance (Bürger and Chory, 2019; Shine et al., 2019), as well as a decrease in salicylic acid, potentially suggesting that pathogen effectors blocked plant defense responses (Giraldo and Valent, 2013). Moreover, campesterol was upregulated in mildew-infected leaves. This metabolite is a precursor to phytohormones (brassinosteroids) (Bürger and Chory, 2019) and could indicate cross-talk to regulate a growth-defense trade off. Similar to findings by Kang et al. (2018), we found that proline levels tended to increase in powdery mildew infected leaves. Proline accumulates in response to abiotic and biotic stresses (Noctor et al., 2015), and increases in proline levels can be triggered by reactive oxygen species (Vaseva et al., 2021), which can act as anti-microbial agents, block pathogen entry at the cell wall, and induce local or systemic defense responses (Lamb and Dixon, 1997; Torres, 2010). Since the duration of infection is likely to have a strong influence on the metabolic responses of the plant (Ward et al., 2010; Tzin et al., 2015), future studies could monitor the metabolic changes in pathogen-challenged plants using a time-series experiment (Kloth et al., 2019). While practically challenging, such studies will give further insights into the metabolic consequences of pathogen infection, such as the upregulation vs. downregulation of defense pathways through time.

Seedlings infested by aphids also had a distinct metabolome compared to healthy seedlings, but compared to mildew-infected seedlings, different metabolites were affected or metabolite levels were affected in opposite directions. Contrary to mildew-infected seedlings, sucrose significantly increased in aphid infested leaves compared to healthy leaves. Upon aphid infestation, leaves may become a local sink for this sugar and other photosynthates (Botha and Matsiliza, 2004; Jakobs et al., 2019), though high sucrose concentrations may reduce aphid performance when the ratio of sugars to amino acids becomes too high (Simpson et al., 1995). Aphid infestation appeared to induce oxidative stress, and the upregulation of several metabolites suggest that the plant protects itself against oxidative damage by detoxifying reactive oxygen species. We observed an increase in metabolites of the arscorbate cycle, i.e., ascorbic acid and dehydroascorbic acid (Smirnoff and Critchley, 2000), as well as an intermediate of their degradation, threonic acid (Noctor et al., 2015), which is crucial for detoxification of reactive oxygen species. Tocopherols and inositols increased, which can act as antioxidants to remove radicals (Loewus and Murthy, 2000; Lushchak and Semchuk, 2012; Płonka et al., 2021). In line with our findings, Ponzio et al. (2017) detected an increase in α-tocopherol levels 72 h after aphid infestation. The production of reactive oxygen species has previously been shown to be induced upon aphid feeding, and can be involved in cell wall restructuring after feeding damage, induction of systemic acquired resistance, and can have a direct toxic effect on aphids by oxidizing proteins in their saliva (Morkunas et al., 2011; Shine et al., 2019). To further disentangle the metabolic responses upon aphid feeding in natural systems, future studies could investigate multiple plant tissues to identify potential changes in source-sink dynamics (Lemoine et al., 2013) as well as local and systemic changes in plant metabolites, e.g., related to defense responses (Marti et al., 2013; Dugé de Bernonville et al., 2017).

Contrary to the changes in metabolic profiles observed after single attacks, the metabolome of dual-attacked leaves did not differ significantly from that of control leaves. Hence, metabolic responses to dual attack could not be predicted from metabolic responses to single attacks when measured at similar time points (72 h after initial attack). Non-additive plant responses as detected in our study have been shown before by studies on multiple abiotic stressors (Rizhsky et al., 2004; Mittler, 2006), as well as for combinations of abiotic and biotic stressors (Atkinson and Urwin, 2012; Suzuki et al., 2014; Pandey et al., 2015). For example, Papazian et al. (2016) found that plant metabolic responses to ozone were counteracted when plants were damaged by herbivores, and as a result, plants exposed to both ozone and herbivory were metabolically indistinguishable from plants only attacked by herbivores. While only few metabolomic studies have focused on multiple biotic interactions, these studies also identified non-additivity in plant responses. Kutyniok and Müller (2012) found that belowground nematodes diminished the leaf metabolic response of *Arabidopsis thaliana* to aphids when attacking simultaneously, indicating crosstalk mechanisms. Ponzio et al. (2017) found that simultaneous attack by caterpillars and aphids on *Brassica nigra* resulted in a metabolic profile similar to that of single attack by caterpillars, suggesting that metabolic responses to the caterpillar outweighed those induced by aphid feeding. While these previous studies investigated dual attackers that putatively induce the JA- and SA-pathway, our study included attackers that both mainly induce the SA-pathway, i.e., aphids and mildew, and reciprocal antagonistic crosstalk was not expected. While speculative, one possible explanation is that non-additive effects on the plant metabolome might have been caused by differences in the concentrations of SA induced upon single vs. dual attack (Koo et al., 2020). For example, SA induces the hypersensitive response with programmed cell death, but at high concentrations—e.g., due to the presence of both attackers—SA can negatively regulate programmed cell death, hence blocking plant defense responses (Radojièiæ et al., 2018). Such a negative feedback mechanism may prevent significant losses of plant tissue when facing substantial attack rates. Another hypothesis that could explain non-additive responses in our experiment, is that dual attack may initiate a faster metabolic response than single attack, which can only be detected when measuring metabolic responses during several time points. To explore the generality of our findings, future studies could investigate the metabolic responses of plants to dual attackers that both induce the SA pathway in other natural and agricultural systems, and contrast these findings to studies that focus on dual attack by organisms that induce the SA- and JA pathway (Kutyniok and Müller, 2012; Ponzio et al., 2017). Moreover, while practically challenging, studies that follow plant metabolic responses to single and dual attack through time will be invaluable to fully understand plant defensive strategies and metabolic changes in response to various combinations of attackers.

## Conclusion

By using GC-MS, we detected distinct metabolic responses of oak seedling leaves upon single attack by mildew or aphids, but not when attacked by mildew and aphids simultaneously, 72 h after attacks were initiated. Moreover, differences in soil microbiomes did not translate into different effects on the leaf metabolome. Our study takes a first step in identifying the molecular consequences of below- and aboveground interactions for oak seedlings in a natural context, i.e., with genetic variation, multiple attackers and natural soil microbiomes. While studies on specific microbial taxa, single attackers or model plant species can link functional responses of plants to specific taxa, studies such as ours are needed to test how such responses play out in a more complex, natural setting. Therefore, we encourage future studies to investigate metabolic responses of plants to below- and aboveground interactions for a diversity of natural study systems and through time, to (i) disentangle the relative importance of below- and above ground interactions on the plant metabolome, (ii) investigate how the plant metabolome changes through time in response to multiple interactions, (iii) elucidate how different plant organs respond to local and systemic interactions, (iv) uncover how plants respond to single and multiple attackers, and identify the presence of potential positive or negative feedback mechanisms, and (v) investigate the diversity in metabolic responses across study systems and identify general patterns. Our results suggest that plant metabolic responses to multiple attackers are interactive, and resolving the molecular mechanisms behind these interactions might thus contribute to our ability to predict the outcomes of plant-pathogen-insect interactions in natural and agricultural settings.

## Data availability statement

The original contributions presented in the study are publicly available. This data can be found here: https://doi.org/10.5061/dryad.vq83bk3w9.

## Author contributions

LD, ER, BA, KP, JE, and AT designed the research. LD conducted the greenhouse experiments. ER and BA conducted the metabolomic work. AA conducted the bioinformatic analyses on soil microbiomes. ER and LD analyzed the data, with help of HS. LD and ER wrote the manuscript, with contributions from all authors.
