# Supplementary material for: Single, but not dual, attack by a biotrophic pathogen and a sap-sucking insect affects the oak leaf metabolome
Source: Front Plant Sci. 2022 Aug 3;13:897186. doi: 10.3389/fpls.2022.897186 (PMC9381920; doi:10.3389/fpls.2022.897186)
Supplement: Supplementary file 1 [file Data_Sheet_1.docx]

**Text S1**. Sequencing methods and bioinformatics of fungal and bacterial communities in the forest and meadow soil (van Dijk et al., in revision).

Quoted from van Dijk et al. (in revision)

*“****Molecular methods***

*DNA extractions were conducted using the DNeasy PowerSoil Kit (Qiagen), following the standard protocol. To identify the fungal taxa, we used tagged primers targeting the ITS2 region, with forward primer ITS3_KYO2 (GATGAAGAACGYAGYRAA) and reverse primer ITS4 reverse (TCCTCCGCTTATTGATATGC). To identify bacterial taxa, we used primers targeting the 16S region, with forward primer 515bF (GTGYCAGCMGCCGCGGTAA) and reverse primer 806 reverse (GGACTACNVGGGTWTCTAAT). Amplification of the ITS2 and 16S regions was verified with a spot-check on a 2% agarose gel. Samples were barcoded to facilitate binding of the DNA to the flowcell (i5 and i7), and barcode incorporation was verified for each sample on a 2% agarose gel. Quantification of each of the amplicons was conducted with the Quant-iT PicoGreen dsDNA Assay Kit (Life Technologies). Libraries were generated by pooling same quantity (ng) of each amplicon. Libraries were cleaned with sparQ PureMag Beads (Quantabio). The libraries were quantified using Kapa Illumina GA with Revised Primers-SYBR Fast Universal kit (Kapa biosystems). Average fragment size was determined using a LabChip GX (PerkinElmer) instrument. Before sequencing, 12% of phix control library was added to the amplicon pool, loaded at a final concentration of 8pM. The amplicon pool was sequenced with the Illumina MiSeq system, using the MiSeq Reagent kit v2 500 cycles (Illumina) and LNA modified custom primers (Exiqon) (primer read 1, LNA-CS1: ACACTGACGACATGGTTCTACA; primer read 2, LNA-CS2: TACGTAGCAGAGACTTGGTCT; primer index read, LNA-CS2rc: AGACCAAGTCTCTGCTACCGTA). All molecular work was conducted by Genome Quebec (Canada).*

***Bioinformatics***

*Sequencing reads were filtered to remove low quality reads, chimeric sequences and sequencing errors. We created amplicon sequence variants (ASVs) using DADA2 asb implemented in phyloseq* (McMurdie and Holmes, 2013)*. When calculating species richness and Shannon diversity, ASV tables were rarefied at an even depth (16S: 11562, ITS: 16982). To evaluate the differences in the microbial community composition among the different soils, we first normalized the ASV tables using cumulative sum scaling CSS method, and then calculated differences between samples using Bray-Curtis dissimilarity metric. We statistically compared microbial community composition among soils using the function adonis2 from the package vegan* (Oksanen et al., 2020)*.”***Text S2.** Detailed protocol of metabolomics analyses, including GC-MS analysis.

**GC-MS analysis**

Derivatization and GC-MS analysis were performed as described previously (Gullberg et al., 2004). 0.5 μL of the derivatized sample was injected in splitless mode by a L-PAL3 autosampler (CTC Analytics AG, Switzerland) into an Agilent 7890B gas chromatograph equipped with a 10 m × 0.18 mm fused silica capillary column with a chemically bonded 0.18 μm Rxi-5 Sil MS stationary phase (Restek Corporation, U.S.) The injector temperature was 270°C, the purge flow rate was 20 mL min^-1^ and the purge was turned on after 60 seconds. The gas flow rate through the column was 1 mL min^-1^, the column temperature was held at 70°C for 2 minutes, then increased by 40°C min^-1^ to 320°C, and held there for 2 minutes. The column effluent was introduced into the ion source of a Pegasus BT time-of-flight mass spectrometer, GC/TOFMS (Leco Corp., St Joseph, MI, USA). The transfer line and the ion source temperatures were 250°C and 200°C, respectively. Ions were generated by a 70 eV electron beam at an ionization current of 2.0 mA, and 30 spectra per second were recorded in the mass range m/z 50 - 800. The acceleration voltage was turned on after a solvent delay of 150 seconds. The detector voltage was 1800 - 2300 V.

For the GC-MS data, all non-processed MS-files from the metabolic analysis were exported from the ChromaTOF software in NetCDF format and imported to MATLAB™ R2016a (Mathworks, Natick, MA, USA), where all data pre-treatment procedures, such as base-line correction, chromatogram alignment, data compression and Multivariate Curve Resolution were performed (Jonsson et al. 2005). The extracted mass spectra were annotated (putatively/tentatively identified) by library comparisons of their retention index and mass spectra (Schauer et al. 2005). Mass spectra and retention index comparison was performed using NIST MS 2.2 software and annotation of mass spectra was based on reverse and forward searches. Both SMC’s in-house standard libraries and the public libraries NIST (https://chemdata.nist.gov/), MoNA (https://mona.fiehnlab.ucdavis.edu/) and MS-DIAL (http://prime.psc.riken.jp/compms/msdial/main.html) were used.

**Table S2.** Summary of the OPLS-DA model to compare metabolomes of oak seedlings that were grown with different soil communities (forest and meadow soil microbial communities). We included one predictive and one orthogonal component to minimize the risk of overfitting the models (Ponzio et al., 2017). The cumulative R^2^ and Q^2^ values of the model are given.

|  | **Components** | **R^2^X(cum)** | **R^2^Y(cum)** | **Q^2^(cum)** |
| --- | --- | --- | --- | --- |
| Forest vs. meadow soil  microbial community | 1+1+0 | 0.208 | 1 | -0.044 |

**Table S3.** Summary of the pairwise OPLS-DA models (treatment A vs. treatment B) to compare metabolomes of oak seedlings that were exposed to different attacker treatments. “Healthy seedlings” refers to seedlings that received no attackers, “Mildew only” to seedlings attacked by powdery mildew, *Erysiphe alphitoides*, “Aphids only” to seedlings attacked by aphids, *Tuberculatus annulatus*, and “Both mildew and aphids” to seedlings attacked by both powdery mildew and aphids. For each model, we included one predictive and one orthogonal component to facilitate model comparisons and to minimize the risk of overfitting the models (Ponzio et al., 2017). The cumulative R^2^ and Q^2^ values of each model are given.

| **Model** | **Treatment A** | **Treatment B** | **Components** | **R^2^X(cum)** | **R^2^Y(cum)** | **Q^2^(cum)** |
| --- | --- | --- | --- | --- | --- | --- |
| 1 | Healthy seedlings | Mildew only | 1+1+0 | 0.272 | 0.876 | 0.216 |
| 2 | Healthy seedlings | Aphids only | 1+1+0 | 0.291 | 0.904 | 0.493 |
| 3 | Healthy seedlings | Both mildew and aphids | 1+1+0 | 0.250 | 0.962 | -0.143 |
| 4 | Mildew and aphids | Mildew only | 1+1+0 | 0.246 | 0.913 | 0.467 |
| 5 | Mildew and aphids | Aphids only | 1+1+0 | 0.302 | 0.907 | 0.276 |

**Table S4.** Overview of metabolites that differed in relative abundance in oak seedlings when attacked by powdery mildew or aphids, compared to healthy seedlings. This table only shows metabolites of special interest (VIP ≥ 1) as derived from the pairwise OPLS-DA models comparing i) healthy seedlings vs seedlings attacked by mildew and ii) healthy seedlings vs seedlings attacked by aphids. Metabolites with lower relative abundance in mildew or aphid attacked seedlings than healthy seedlings are indicated with a “-”, and metabolites with higher abundance in mildew or aphid attacked seedlings are indicated with a “+”. The last two columns present metabolites that changed in relative abundance in response to attack by mildew as well as aphids, where metabolites that were affected in similar directions or opposite directions are presented in separate columns. For the column that presents metabolites that were affected in opposite directions by mildew and aphids, we first present the directionality of the effect for mildew, then for aphids (i.e. mildew / aphids). Stars indicate if the relative abundance of the metabolite significantly differed between seedlings attacked by mildew only or aphid only and healthy seedlings (* = p < 0.05, ** = p < 0.01, and *** = p < 0.001, from linear models, function lm, R v.3.6.1 (R Core Team, 2020)). For further information on which treatment groups differ, see Fig. S4. Metabolites are listed in descending order of VIP value.

| **Metabolites induced upon attack by:** | | | |
| --- | --- | --- | --- |
| **Mildew** | **Aphids** | **Mildew and aphids, in a similar direction** | **Mildew and aphids, in opposite directions** |
| Glucose (-) * | Palmitoleic acid (+) ** | Arabinose (-) | Succinic acid (- / +) ** |
| Fructose (-) | scyllo-Inositol (+) ** | Glucaric acid (+) | Sucrose (- / +) * |
| Levoglucosan (-) | p-Coumaric acid (+) ** | Hexose (-) | Malic acid (- / +) |
| Gallic acid (-) | Dehydroascorbic acid (DHAA) (+) |  | |
| Salicylic acid (-) | chiro-Inositol (+) * |  |  |
| Glucose 1-phosphate (-) | gamma-Aminobutyric acid (GABA) (-) |  |  |
| Erythritol (-) | Ascorbic acid (+) |  |  |
| Kaempferol (-) | myo-Inositol (-) |  |  |
| Uridine diphosphate glucuronic acid (-) | gamma-Tocopherol (+) |  |  |
| Campesterol (+) | alpha-Tocopherol (+) * |  |  |
| Serine (+) * | 2-Ethylhexanoic acid (+) |  |  |
| beta-Sitosterol (+) | Threonic acid (+) |  |  |
| Proline (+) | Quinic acid (+) |  |  |
| Catechin (+) | Myo-Inositol 1-Monophosphate (-) |  |  |
| Cycloartenol (+) | Phosphate (-) |  |  |
| Pipecolic acid (-) | UHMOGBXQYJLCHX-IMSYWVGJSA-N (-) |  |  |
| Glyceric acid (-) | Maleic acid (+) |  |  |
| Ribose (-) |  |  |  |

**Figure S1.** Setup and design of the greenhouse experiment, including the **(A)** soil microbiomes, **(B)** attacker treatments, and **(C)** timeline of the preparations and the experiment. Panel **(A)** shows the preparation of the two different soil biotas. The forest soil microbiome was created by mixing forest soil with sterilized meadow soil and sterilized soil of a third soil type in equal parts (1:1:1), i.e. the “forest soil mix”. The meadow soil microbiome was created by mixing meadow soil with sterilized forest soil and sterilized soil of a third soil type in equal parts (1:1:1), i.e. the “meadow soil mix”. Mixing with sterilized soils was done to ensure that the biotic communities but not abiotic components of the soil mixes differed (left). Then, pots were filled with sterilized potting soil, one of the natural soil mixes (either forest soil mix or meadow soil mix) and a top layer of sterilized sand (right). Panel **(B)** shows the different attacker treatments to which oak seedlings were exposed, including: 1) Healthy seedlings, which received no attackers (orange), 2) mildew only (blue), 3) aphids only (green), and 4) both mildew and aphids (pink). Leaf samples of seedlings were collected 72 hours after introduction of attackers. Panel **(C)** shows the timeline preparations with regards to soil collection and seedling growth, and the experimental steps.

*
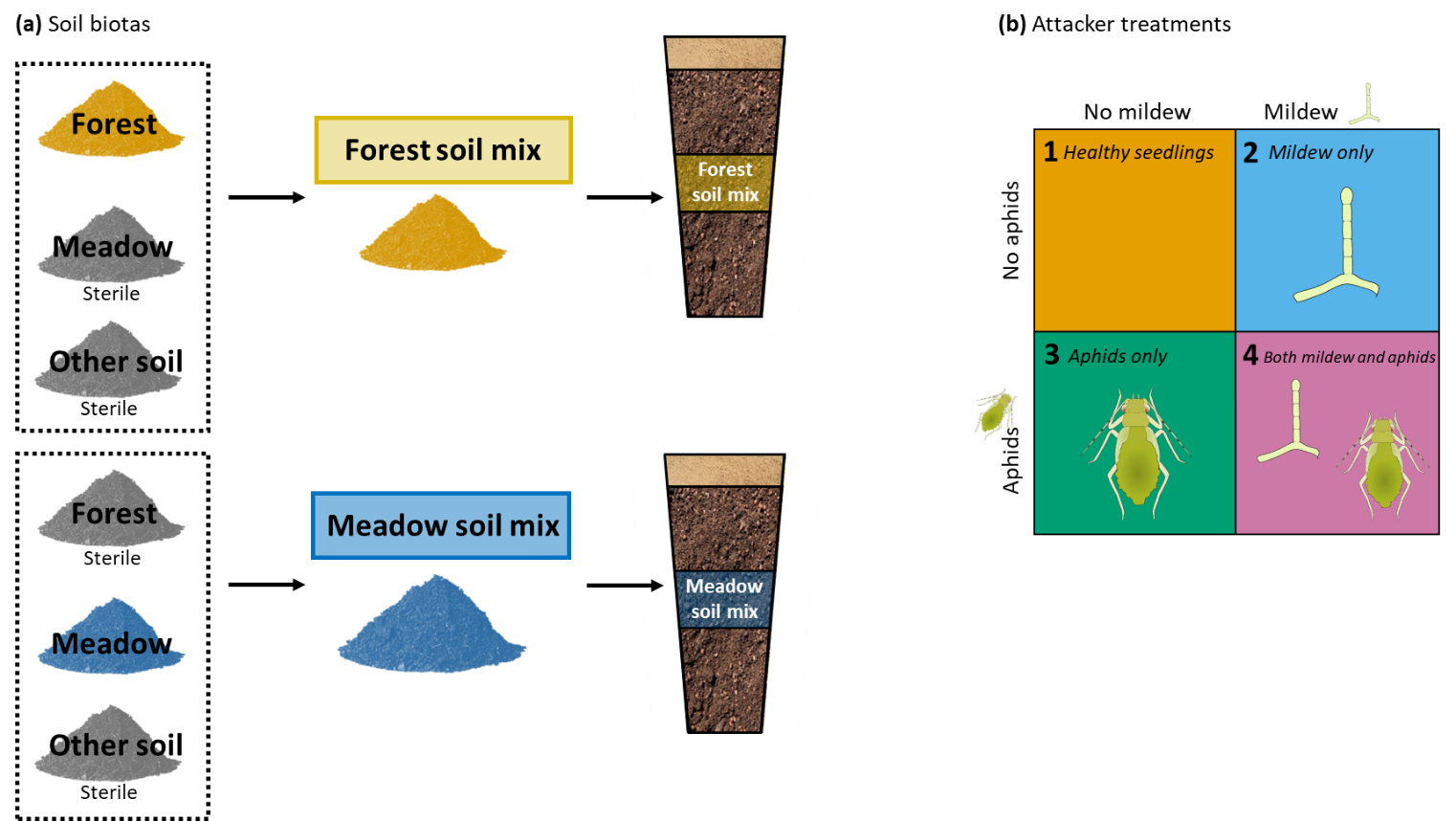
*

*
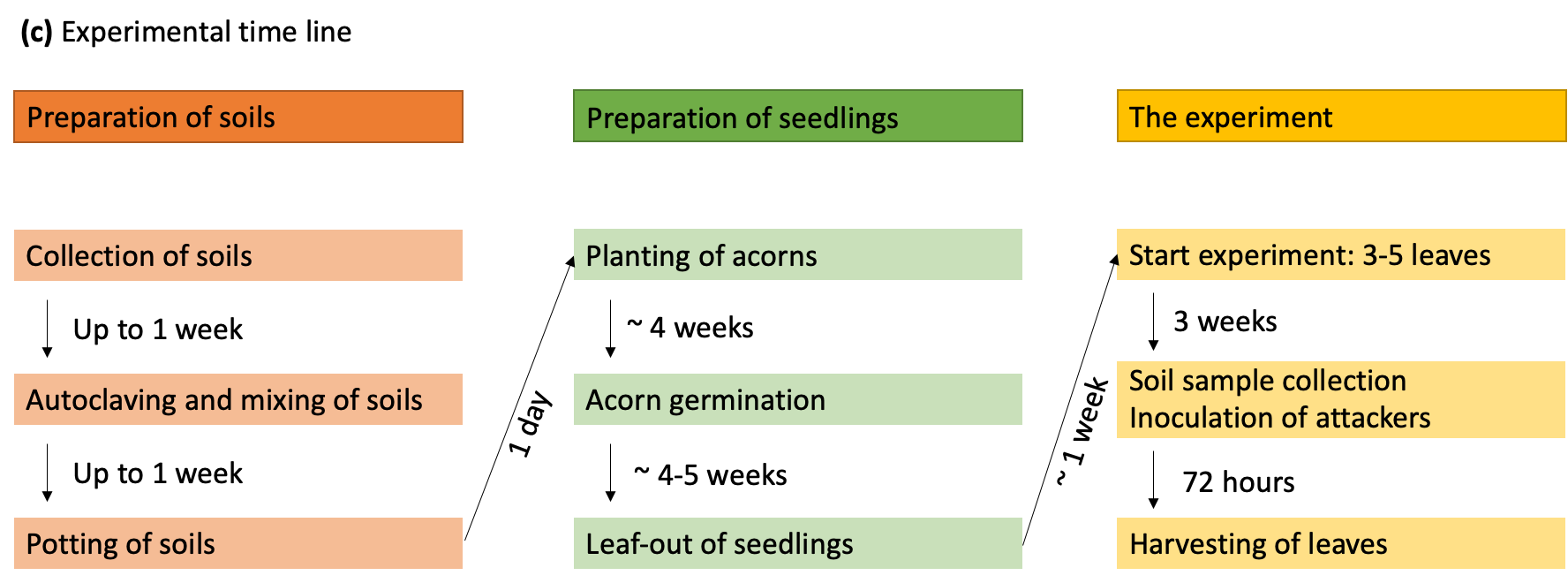
*

**Figure S2.** Bacterial and fungal communities in forest and meadow soils. Panel **(A)** shows bacterial community composition in forest and meadow soils, and panels **(B)** and **(C)** present the relative abundance (scaled with circle size) of bacterial phyla in the forest and meadow soil, respectively. Panel **(D)** shows fungal community composition in forest and meadow soils, and panels **(E)** and **(F)** present the relative abundance (scaled with circle size) of fungal phyla in the forest and meadow soil, respectively. If a taxon is presented as “uncultured”, sequences were present in the database, but matched to an uncultured taxon. If a taxon is “unidentified”, sequences were not present in the database. Visualizations of community compositions are based on NMDS ordination with Bray-Curtis distance matrix.


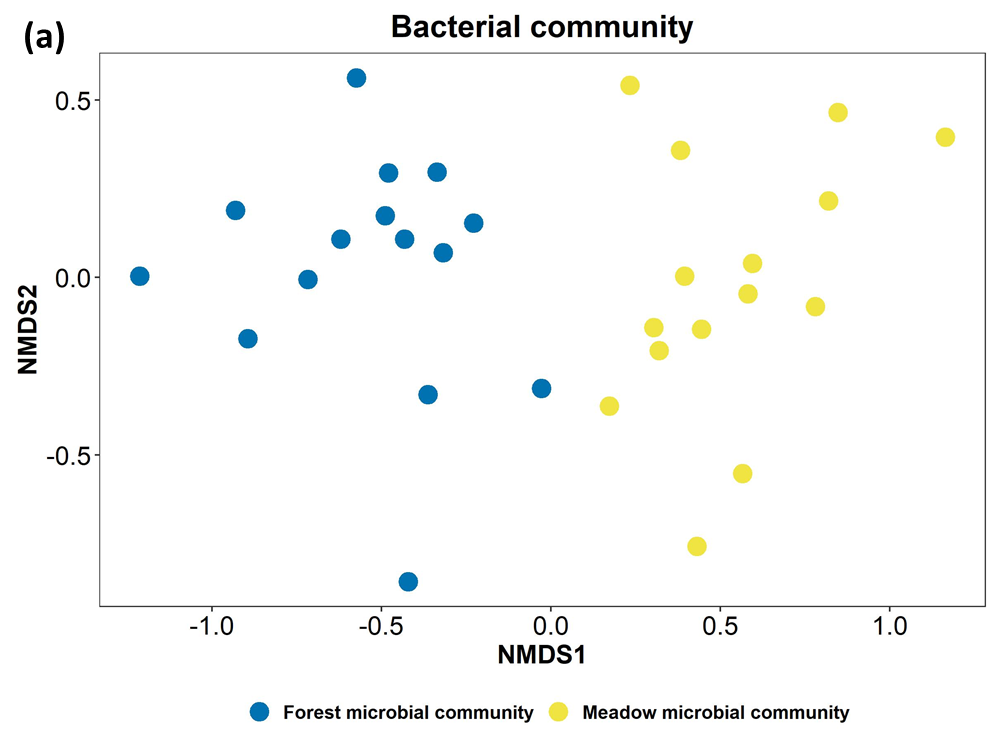


**
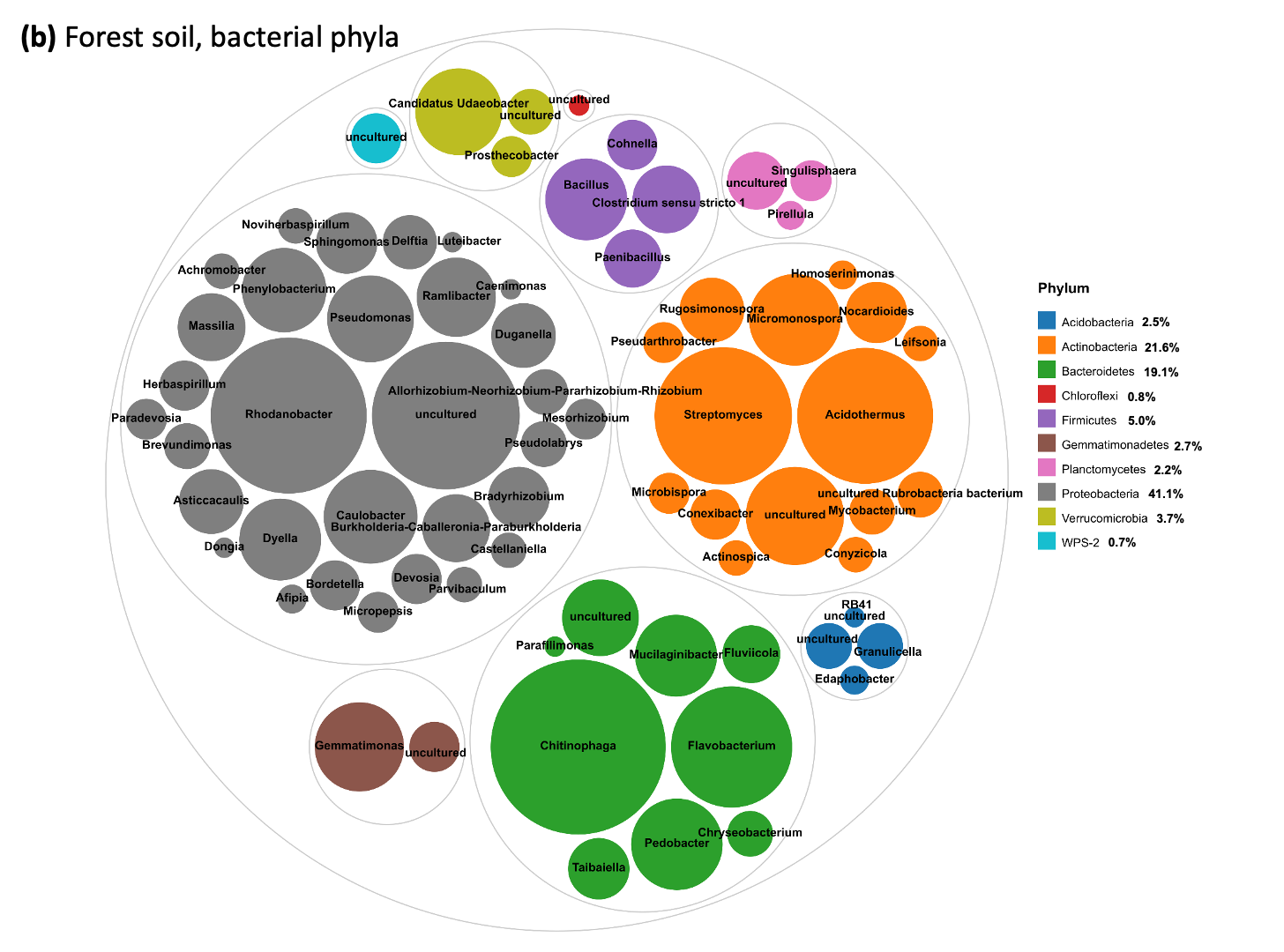
**

**
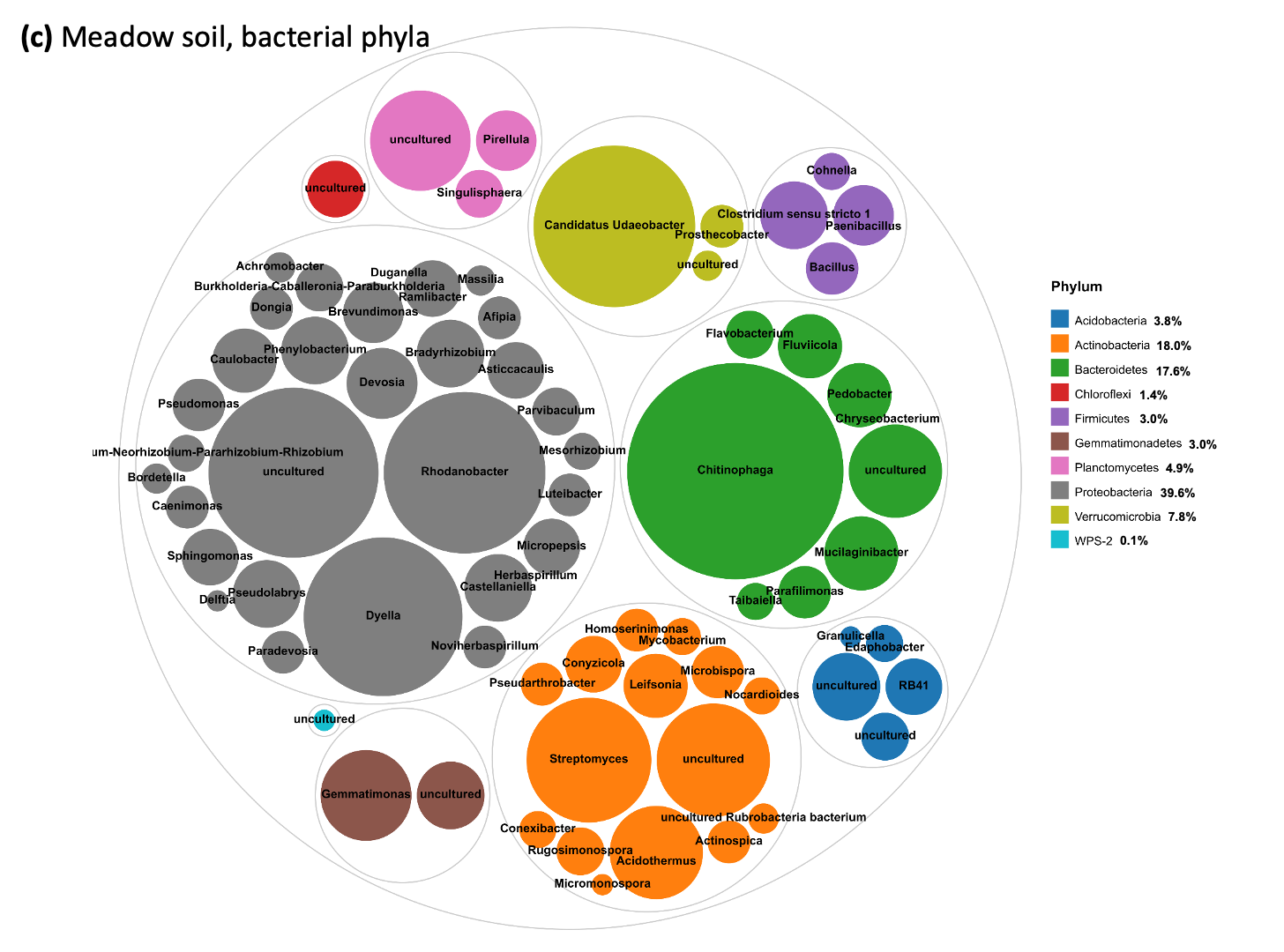
**

**
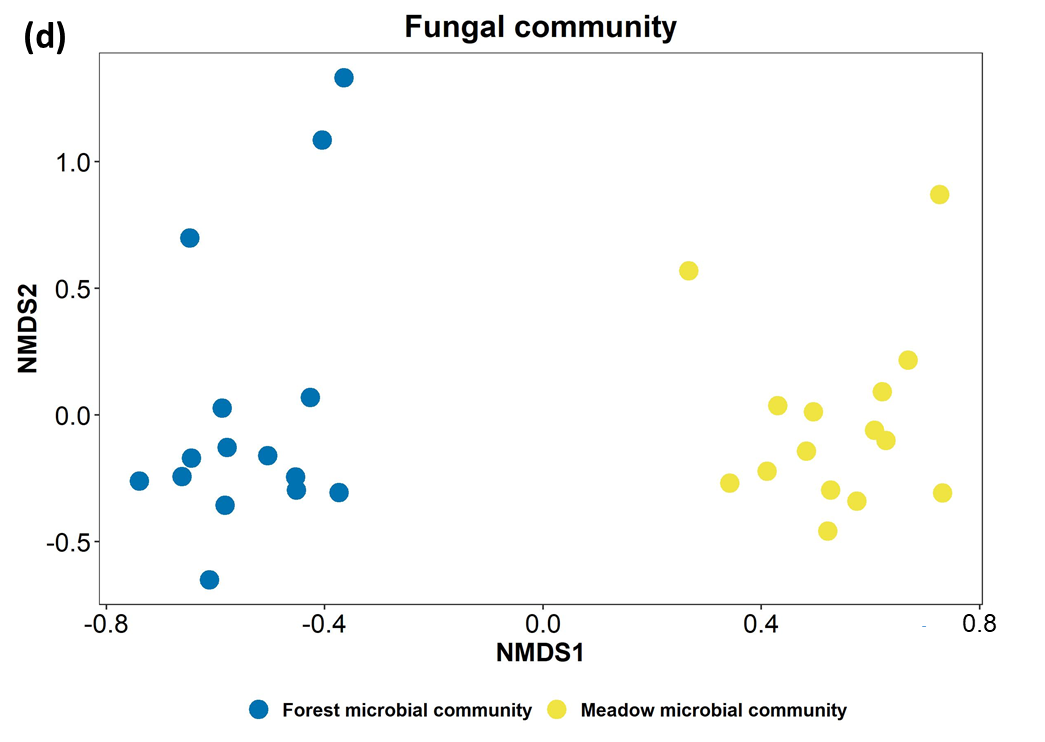
**

**
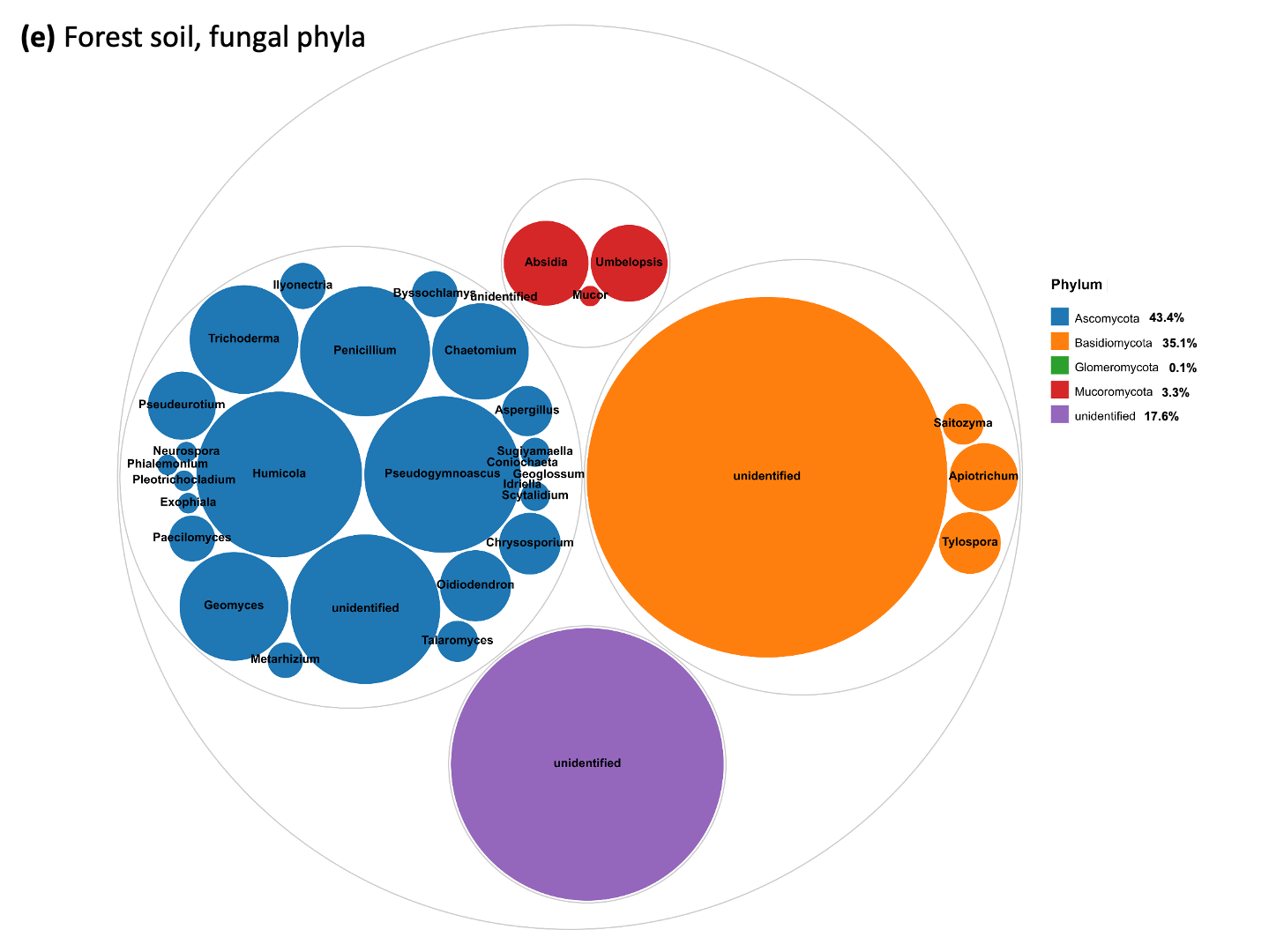

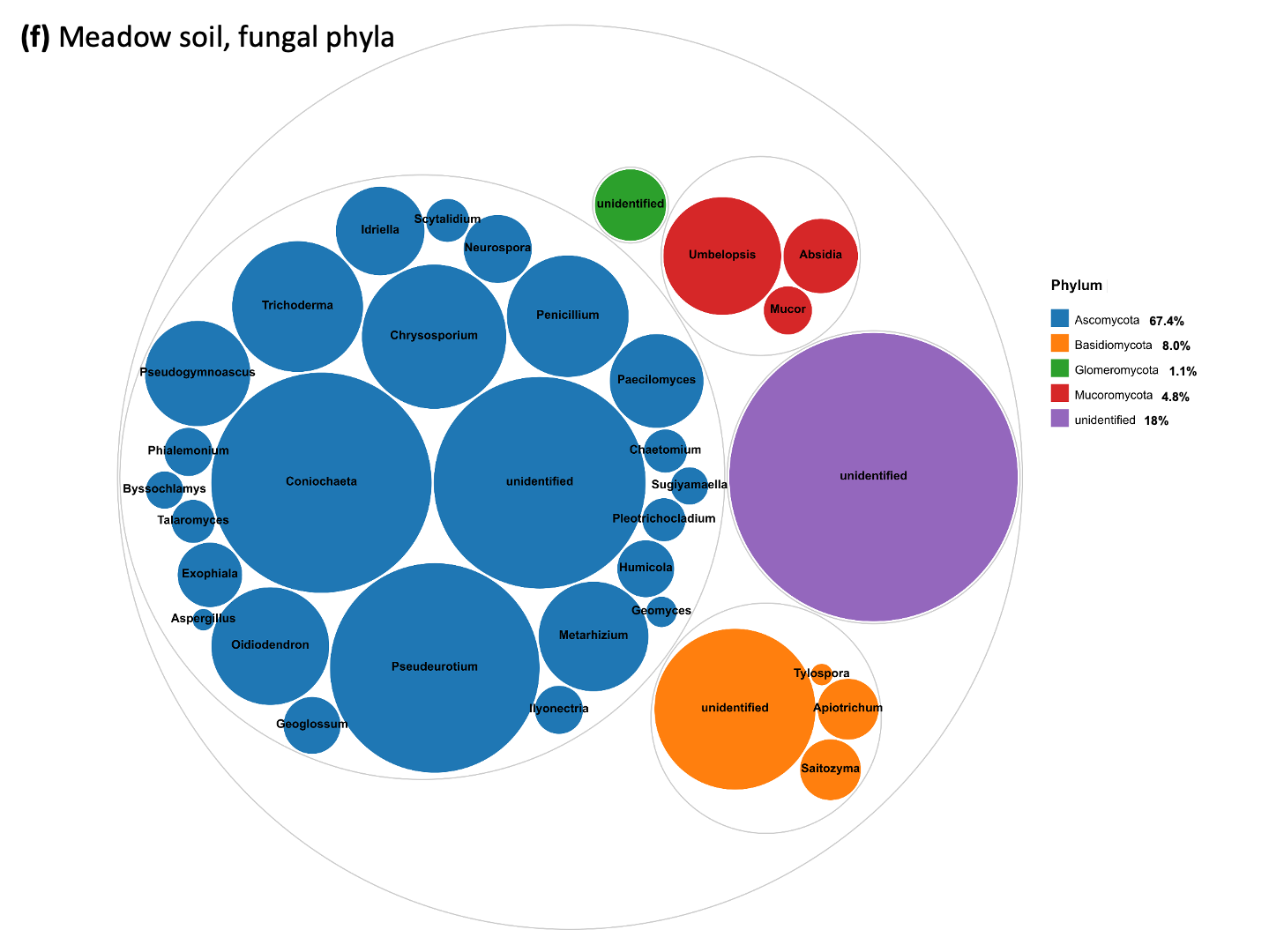
 Figure S3.** Changes in the metabolic profiles of oak seedlings in response to attackers, visualized in a score plot with the first two components of the OPLS-DA model. Colours represent the different attacker treatments, including: 1) Healthy oak seedlings (orange), 2) Seedlings attacked by mildew (blue), 3) Seedlings attacked by aphids (green), and 4) Seedlings attacked by mildew and aphids (pink), with 8 replicates per treatment. The model had a total of three predictive components and one orthogonal component, with a cumulative R^2^X of 0.448 (and a predictive R^2^X of 0.308) and a cumulative Q^2^ of 0.238. The first two predictive components explained 26.2% (R^2^X, 0.164 and 0.098 respectively) and 66.7% (R^2^Y, 0.333 and 0.333 respectively) of the variation in metabolic profiles, and the model had a predictive power of 0.148 (Q^2^, 0.099 and 0.050 respectively).


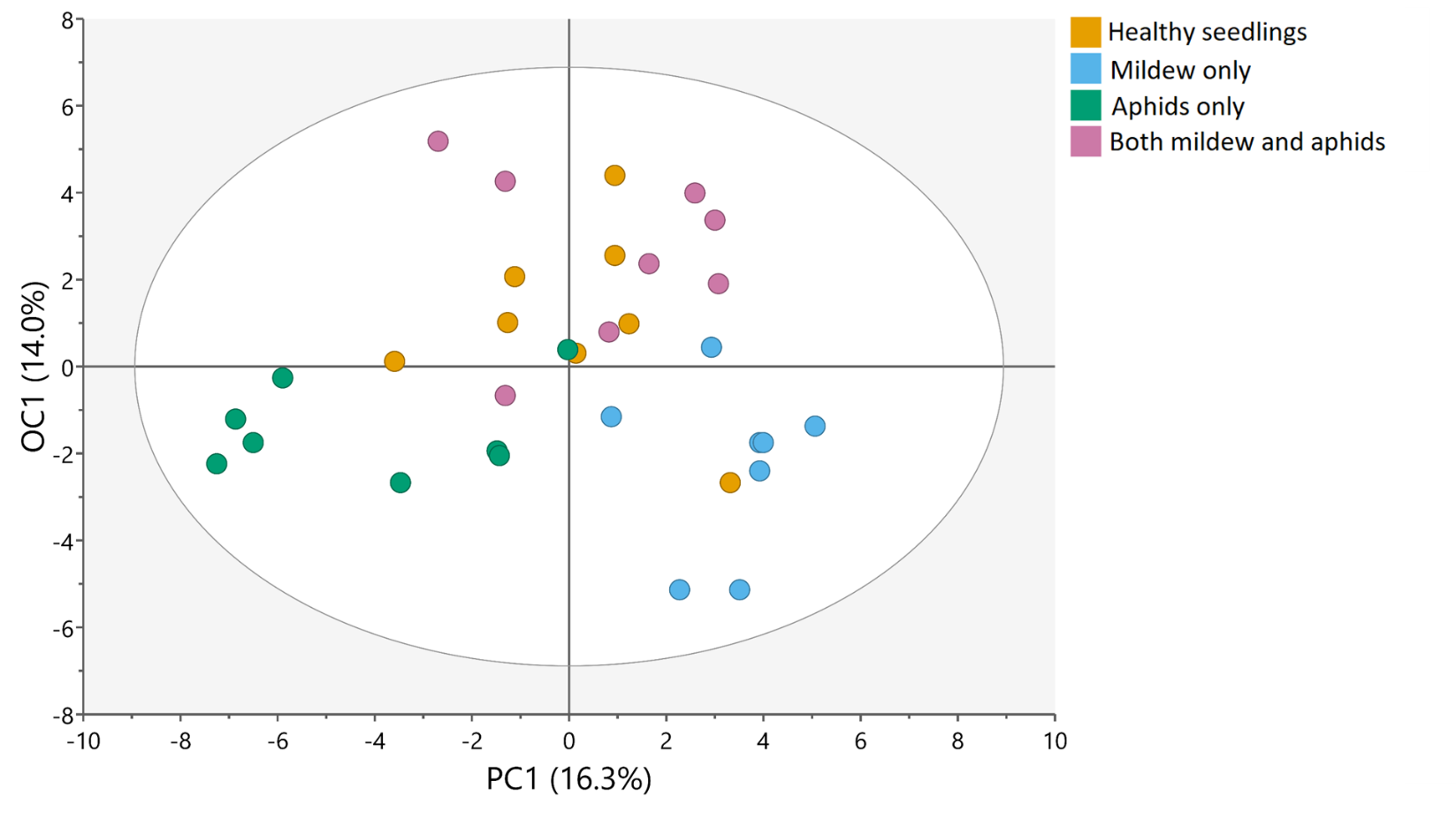


**Figure S4.** SUS-plots of **(A)** control vs. pathogen and control vs. aphid, **(B)** pathogen vs. control and pathogen vs. both and **(C)** aphid vs. control and aphid vs. both. In each panel, the axes of the SUS-plots are the p(corr) values of two pairwise OPLS-DA models (see Table S3 for an overview of the full set of OPLS-DA models). Panel **(A)** shows how metabolite levels differ between mildew infected seedlings and aphid infested seedlings (with healthy seedlings as the shared treatment). The p(corr) values (scaled loadings as correlation coefficients between the model and the data) from Model 1 are shown on the abscissa, and those from Model 2 on the ordinate. Panel **(B)** shows how metabolite levels differ between healthy seedlings and seedlings attacked by both mildew and aphids (with mildew infected seedlings as the shared treatment). The p(corr) values from Model 1 are shown on the abscissa, and those from Model 4 on the ordinate. Panel **(C)** shows how metabolite levels differ between healthy seedlings and seedlings attacked by both mildew and aphids (with aphid infested seedlings as the shared treatment). The p(corr) values from Model 2 are shown on the abscissa, and those from Model 5 on the ordinate.


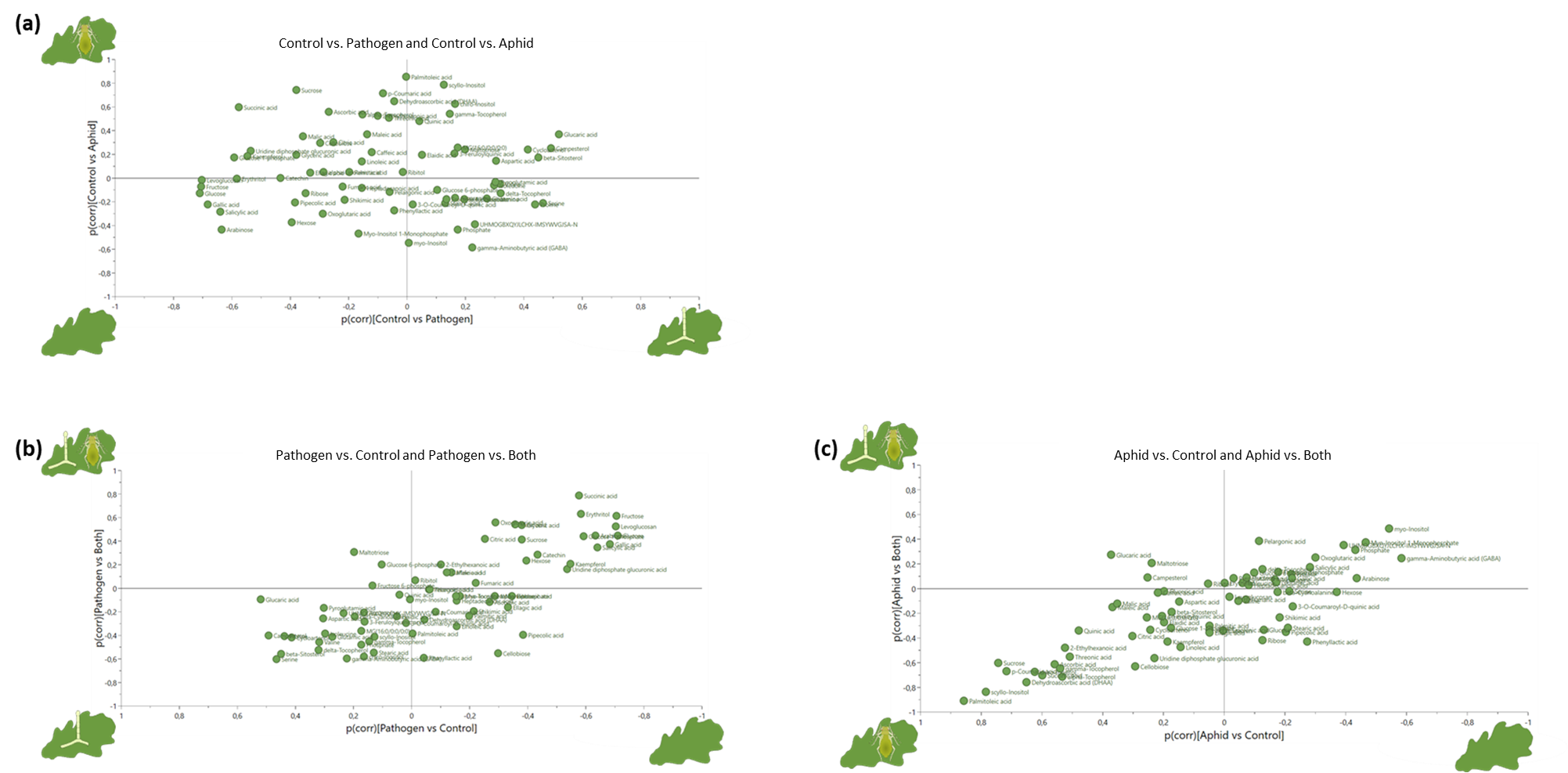


**Figure S5.** Boxplots showing the effect of attacker treatment on the metabolite concentration for metabolites of special interest (with VIP ≥ 1). There were eight replicates per treatment group, and each treatment is represented by a different colour (healthy seedlings as orange, seedlings attacked by mildew only as blue, seedlings attacked by aphids only as green, and seedlings attacked by both mildew and aphids as pink). The vertical axis is the relative abundance of the metabolite after normalization. Effect of attacker treatment was evaluated by one-way ANOVAs for models with normally distributed residuals, or otherwise Kruskal-Wallis tests, and pairwise comparisons were conducted using Post-Hoc Tukey’s Honest Significant Differences or Post-Hoc Dunn tests, respectively. Significant p-values (< 0.05) are shown in the top right of the plots. Significant pairwise differences between attacker treatments are indicated by a letter above the box plots, where treatments with the same letter do not differ significantly. Abbreviations used: DHAA = Dehydroascorbic acid, GABA = gamma-Aminobutyric acid, myo-Ins 1-MonoP = myo-Inositol 1-Monophosphate, UDGA = Uridine diphosphate glucuronic acid, UH[...]-N = UHMOGBXQYJLCHX-IMSYWVGJSA-N.

a

b

b

b

a

b

ab

ab

a

b

ab

ab

a

b

ab

b

a

b

ab

ab

a

b

ab

ab

a

b

ab

ab

a

b

ab

ab

p=0.003

p=0.02

p=0.004

p=0.03

p=0.01

p=0.01

p=0.04

p=0.02

a

ab

b

b

a

b

ab

ab

a

b

b

b

a

b

ab

a

b

ab

b

a

b

b

b

b

p<0.001

p=0.01

p=0.03

p=0.005

p=0.04

p=0.001

p=0.01

P<0.001

b

b

b

a

**Figure S6.** Changes in the metabolic profiles of oak seedlings in response to soil microbial communities and attacker treatments, visualized as a loading plot for the first two components of PCA model. The loading plot shows the contribution of individual metabolites to the differences in metabolic profiles for seedlings growing with different soil microbial communities and receiving different attacker treatments. For score plots of the PCA models, see Figure 1.


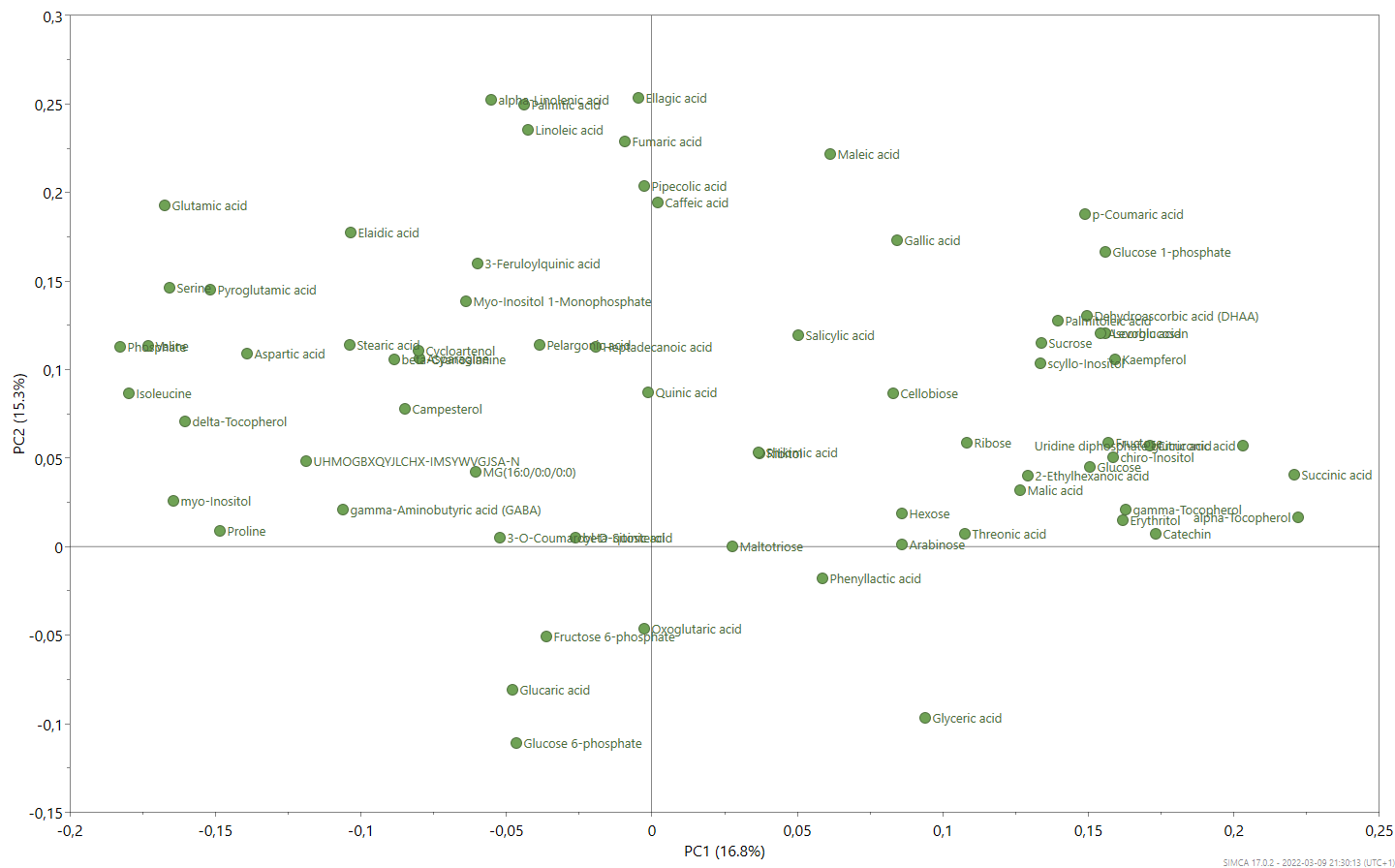


**References**

Gullberg, J., Jonsson, P., Nordström, A., Sjöström, M., and Moritz, T. (2004). Design of experiments: an efficient strategy to identify factors influencing extraction and derivatization of Arabidopsis thaliana samples in metabolomic studies with gas chromatography/mass spectrometry. *Anal. Biochem.* 331, 283–295. doi: 10.1016/j.ab.2004.04.037.

McMurdie, P. J., and Holmes, S. (2013). phyloseq: An R package for reproducible interactive analysis and graphics of microbiome census data. *PLoS ONE* 8, e61217.

Oksanen, J., Blanchet, F. G., Friendly, M., Kindt, R., Legendre, P., McGlinn, D., et al. (2020). *vegan: Community Ecology Package*. Available at: https://CRAN.R-project.org/package=vegan.

Ponzio, C., Papazian, S., Albrectsen, B. R., Dicke, M., and Gols, R. (2017). Dual herbivore attack and herbivore density affect metabolic profiles of *Brassica nigra* leaves. *Plant Cell Environ.* 40, 1356–1367. doi: 10.1111/pce.12926.

R Core Team (2020). *R: A language and environment for statistical computing*. Vienna, Austria. Version 3.6.3: R Foundation for Statistical Computing.

van Dijk, L. J. A., Abdelfattah, A., Ehrlén, J., and Tack, A. J. M. (in revision). Belowground microbiomes drive aboveground plant-pathogen-insect interactions. *Revis.*
